# Concurrent Measurement of Local and Global in-Plane Permeability of Reinforcement Fabrics through Real-Time Image Processing

**DOI:** 10.3390/polym15153233

**Published:** 2023-07-29

**Authors:** Bin Yang, Jihui Wang, Yuwei Feng, Mingfan Zhan, Aiqing Ni

**Affiliations:** 1 School of Materials Science and Engineering, Wuhan University of Technology, Wuhan 430070, China; yangbin@whut.edu.cn (B.Y.); jhwang@whut.edu.cn (J.W.);; 2 State Key Laboratory of Advanced Technology for Materials Synthesis and Processing, Wuhan University of Technology, Wuhan 430070, China

**Keywords:** reinforcement fabric, local in-plane permeability, spatial variability, real-time monitoring, concurrent measurement

## Abstract

Properties of reinforcement fabrics, such as permeability, are typically characterized in a volume-averaging sense, whereas the fabric microstructure may vary spatially. This makes designing an effective resin infusion strategy for defect-free composite fabrication challenging. Our work presents a concurrent method for simultaneously measuring the local and global in-plane permeability and offers a handy technique for evaluating spatial variability. This experimental setup was similar to that of unidirectional in-plane permeability tests. The fabric, however, should be cut and tested along the angle bisector of warp and weft directions. The evolution of resin flow fronts was analyzed in real-time using in-house code through live video monitoring. The local and global in-plane permeability components were then obtained by applying Darcy’s law regionally and globally. The results are in good agreement with those obtained by radial permeability experiments. Statistical analysis of local permeability reveals that the microstructure variability follows a normal distribution. A complete description of fabric microstructure provided by X-ray microcomputed tomography suggests that local permeability and microstructure variation are closely related, confirming the efficacy of the newly proposed method. This work enables the estimation of fabric structure variability and local and global in-plane permeability in a single test without resorting to expensive volume imaging techniques.

## 1. Introduction

Engineering textiles are widely used in various industrial sectors due to their unique properties, such as high strength, durability, and resistance to chemical substances. Among its many applications is that of a reinforcement fabric in composite materials [1]. The fabrication of composite parts can be achieved through various methods, including liquid composite molding (LCM) [2,3] and pre-impregnated prepregs [4]. The former is a family of advanced composite manufacturing processes that involve the infusion of liquid resin into a preformed fibrous material such as carbon, glass, or natural fabrics, resulting in the formation of a composite structure. On the other hand, in the pre-impregnation method, the fibrous material is pre-impregnated with resin, which is then cured to form the composite structure. Regardless of the method used, resin flow in the reinforcement fabrics is an essential part of the composite manufacturing process to achieve full impregnation. As a result, the permeability of reinforcement fabrics, which is defined by Darcy’s law [5] to reflect the capability of porous material to allow fluid pass through, has a direct impact on the design of the manufacturing process. This is critical for advancing the understanding of manufacturing defect formation mechanism and improving the design and fabrication of high-performance composites.

The measurement of permeability, especially the in-plane components, has received substantial attention in the past 30 years [6,7,8,9]. Despite the straightforward definition, characterization of in-plane permeability has been challenging because the microstructures of textiles can be altered during the woven [10], preforming [11], or infusion processes [12]. This results in poor reproducibility of permeability. This was evidenced by the first international benchmark exercise [6], which showed significant inconsistency among participants regarding the measured in-plane permeability. The error was up to an order of magnitude. Such inconsistency highlights the need for standardized measurement techniques to ensure that in-plane permeability can be measured accurately.

In response to this need, guidelines were specified in the second benchmark exercise [7] for the unidirectional test method tests (also known as the channel flow experiment [13]). The results showed that with the guidelines in place, good consistency (standard deviation of 20%) was achieved among the participants. The standard deviation was further reduced when the Least Square Fit (LSF) method proposed by Ferland et al. [14] was applied for data analysis. Additionally, as presented by May et al. [15], the radial injection method was also shown to have good reproducibility when operation guideline was specified. It allows for two principal components of in-plane permeability and the orientation of the flow ellipse to be characterized simultaneously [16]. It is evident from these findings that standardizing the measurement techniques for permeability characterization can lead to better reproducibility and accuracy.

Spatial variability of permeability caused by the variation in material microstructure is, however, rarely addressed [17]. Instead, conventional test methods utilize only a constant, namely the global permeability, to represent the averaging ability of a fabric to allow liquid to pass through. Spatial variation can, however, lead to a non-uniform flow of resin, which is a major contributing factor to the formation of voids due to gas (either the residual air or resin vapor) entrapment [18]. Various auxiliary equipment, such as linear direct current sensing system [19,20], setups that control the infusion [18,21,22,23], or post-filling [24] processes, have been developed to improve flow uniformity by controlling resin flow actively. Yet, there is still room for improvement in the effectiveness and practicality of these devices in actual production, particularly for large and complex structures.

In fact, depending on the spatial variability of the fabrics, a process design can be optimized by simply adding injection ports or vents within the mold (“passive” control [25]) to prevent incomplete saturation or resin excess. Intuitively, two methods can be adopted to characterize the spatial variability of textiles. One is through experiments such as multiple sampling and permeability testing with the abovementioned methods. This, however, can be labor-intensive. Furthermore, the large sample area may result in an averaging effect that obscures the true variability of a fabric. The other approach uses volume imaging techniques, such as X-ray micro-computed tomography (Micro CT), to observe the microstructure [26]. Nevertheless, the field of imaging is limited (typically on the order of several millimeters [27]) and may not be representative either. On the other hand, multiple scans increase not only the time required but also the cost.

Hence, this study aims to develop a quantitative method to characterize the spatial variability of permeability, which reflects the spatial variations in fabric structure. The method can characterize the local and global in-plane permeability in a single test. Thus, spatial variability can be obtained indirectly without resorting to expensive volume imaging techniques. It is verified by microscale observations of the fabric structure using the non-destructive X-ray computed tomography technique, which allows the fabric structure to be analyzed directly. Moreover, by using a special method of sample preparation, the two-principle in-plane permeability components are obtained simultaneously.

The remainder of this paper is organized as follows: The theory of the proposed method is presented in Section 2, where the underlying principles and assumptions are explained in detail. Section 3 describes the materials and methods used in this experimental investigation. Finally, in Section 4, the obtained results are presented and discussed. We validate the results by comparing them with experimental data obtained from Micro CT. This method provides an easily implemented method to characterize the spatial variability of in-plane permeability. This knowledge can be used to guide the design of the resin infusion strategy to ensure proper flow and wetting of the reinforcement fabrics, ultimately leading to improved quality and performance.

## 2. Theory

In-plane permeability K ($m^{2}$) characterizes the resistance of a fibrous preform to a liquid flow in the in-plane direction. It can be experimentally obtained according to one-dimensional Darcy’s law [5], which reads

(1) $$v=-\frac{K}{\mu \phi }\cdot \nabla p$$

where v is the velocity (m/s) of the flow front; p is the pressure drop (Pa) from the injection port to the flow front, and ∇p represents the pressure gradient (Pa/m). μ is the fluid viscosity (Pa·s), and ϕ is the porosity of the preform.

Assuming the test liquid for in-plane permeability is incompressible and the porosity behind the flow front is constant and fully saturated, the continuity equation can be expressed as follows:

(2) ∇⋅v=0

For 2D flow, it is equivalent to

(3) $$\frac{\partial v_{x}}{\partial x}+\frac{\partial v_{y}}{\partial y}=0$$

where $v_{x}$ and $v_{y}$ are the velocity components in principle directions of x−y plane. In the case of one-dimensional scenarios, the second term on the left-hand side of the equation vanishes.

### 2.1. Global and Local Permeability

The unidirectional permeability test typically measures only the global permeability along the test direction, which represents the overall ability of a porous material to permit liquid to flow through it. It describes the bulk permeability of the material and is averaged over the sample area. In this work, we propose the concept of local in-plane permeability, which refers to the permeability of a small, localized region within a larger porous material. In the case of textile fabrics, local permeability would refer to the permeability of a small section of the fabric rather than the overall (global) permeability of the entire textile sample. Thus, local permeability can vary due to spatial variations in the pore structure and geometry at a small scale.

In this study, the conventional unidirectional test method was used for global in-plane permeability characterization. The pressure drop between the inlet and the flow front remains constant throughout this experiment. The global in-plane permeability, $K_{g}$, was calculated from the measured flow front position $x_{f}$ (m) at time t (second) as follows:

(4) $$K_{g}=\frac{\mu \phi }{2p}\frac{x_{f}^{2}}{t}$$

For the unsaturated unidirectional test, $0<x_{f}<L$. L is the length of the sample. The position of injection inlet $x_{inlet}=0$. It is necessary to record at least three pairs of $\left(t,x_{f}\right)$ during the entire infusion process to enhance permeability measurement reliability. Then Lest Square Fitting was performed to estimate the value of $x_{f}^{2}/t$ and calculate the global in-plane permeability.

The calculation of local permeability requires the knowledge of the transient velocity of the flow front, which can be expressed as the differential of flow front position with respect to time:

(5) $$v=\frac{dx_{f}}{dt}$$

Substituting Equation (3) into Equation (1) and then integrating over a time interval $\left[t_{1},t_{2}\right]$, the local in-plane permeability at the region can be obtained as follows:

(6) $$K_{l}=\frac{\mu \phi }{2p}\frac{x_{f2}^{2}-x_{f1}^{2}}{t_{2}-t_{1}}$$

where $x_{1}$ and $x_{2}$ are the positions (m) of flow front at time $t_{1}$ and $t_{2}$. The spatial distribution of the permeability caused by the fabric geometry complexity can then be quantified by the variability of local in-plane permeability values.

### 2.2. Unidirectional Flow of the Principal Directions

**Figure 1:**
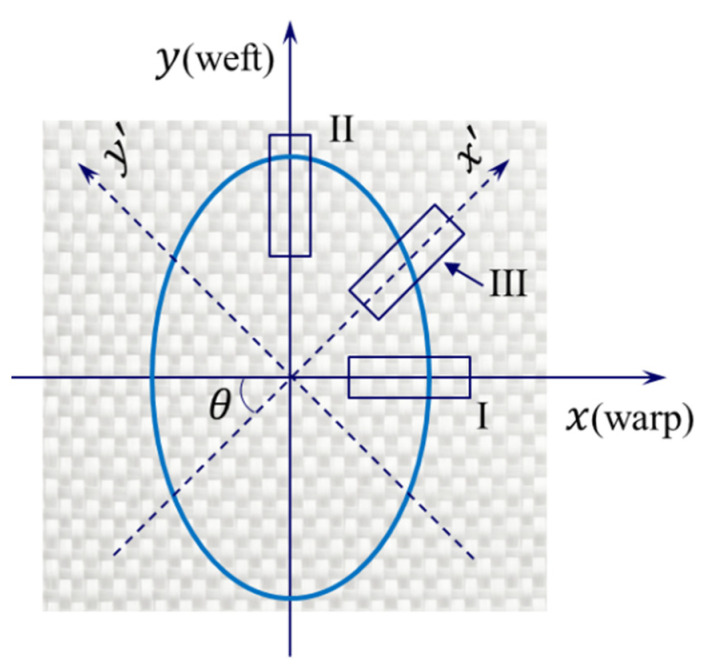
Possible sampling methods for measuring in-plane permeability by the unidirectional method and the permeability ellipse. The x and y directions coincide with the principal directions of the textile.

As illustrated in Figure 1, a unidirectional in-plane permeability test can be conducted along one of the principal directions of the textile (indicated by I and II) or any direction at an angle θ to the x-axis (indicated by III). The in-plane permeability measured from the unidirectional test with a sample taken at θ is denoted by $K_{\theta }$ (also known as effective permeability [28]). Specifically, we denote $K_{0}$ and $K_{90}$ with $K_{x}$ and $K_{y}$ to indicate that they are the principal permeability components. Bear [29] shows that the directional permeability $K_{\theta }$ varies with respect to the angle θ for anisotropic porous media, and its square root conforms to the shape of an ellipse. This is known as the permeability ellipse and is illustrated in blue in Figure 1. Moreover, $K_{x}$ and $K_{y}$ represent the square of the semi-major and semi-minor axes of the permeability ellipse.

**Figure 2:**
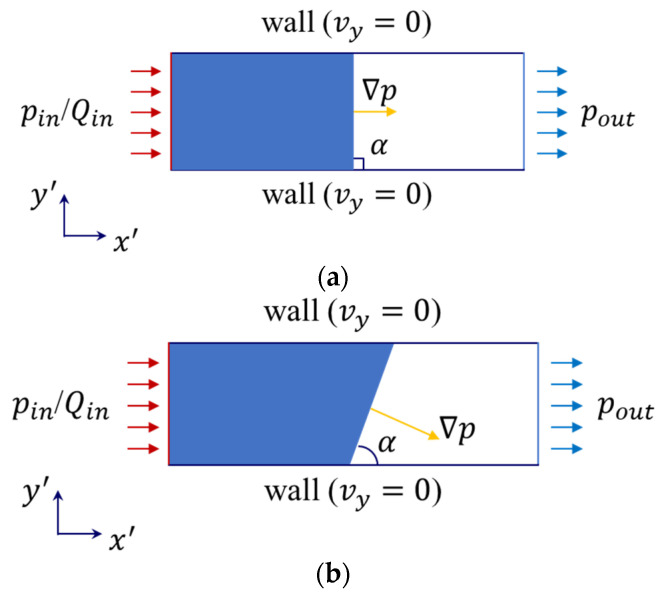
The flow front and its angle with respect to the flow direction ($x^{\prime }$ axis) for anisotropic samples: (**a**) test along the principal direction of textiles (θ=0 or 90° ); (**b**) test off the principal directions (θ≠0 or 90° ).

For the test with the sample along the principal direction (x or y), the pressure gradient follows the direction of flow, and the flow front is perpendicular to the test direction (see Figure 2a, flow front angle α=90 °). However, there may appear to be an inconsistency between the direction of transient pressure and velocity gradients for anisotropic materials when θ≠0° or 90°. In this case, the resin flow front forms a constant flow front angle α<90° with the $x^{\prime }$ axis as the flow stabilizes. This is depicted in Figure 2b. Both flow behaviors can be expressed in a general form using the following equations:

(7) $$\left[\begin{array}{c} v_{x^{\prime }}\\ v_{y^{\prime }} \end{array}\right]=-\frac{1}{\mu }\left[\begin{array}{cc} K_{x^{\prime }x^{\prime }} & K_{x^{\prime }y^{\prime }}\\ K_{x^{\prime }y^{\prime }} & K_{y^{\prime }y^{\prime }} \end{array}\right]\left[\begin{array}{c} \frac{\partial p}{\partial x^{\prime }}\\ \frac{\partial p}{\partial y^{\prime }} \end{array}\right]$$

For sampling along θ=0 or 90°, there is no flow in the $y^{\prime }$ direction, namely,

$$\frac{\partial p}{\partial y^{\prime }}=0\;$$

and

$$v_{y^{\prime }}=0$$

Thus, Equation (5) is reduced to the one-dimensional Darcy’s law. In this case, the principal permeability ($K_{x}$ or $K_{y}$) can be obtained according to Equation (2). In case of θ≠0 or 90°, all four components of the two-dimensional permeability tensor must be known to provide a thorough description of the flow front. The global directional in-plane permeability $K_{\theta }$, on the other hand, provides only an approximation of the flow behavior and fails to consider the potential inclination of the flow front.

The principal permeability components can be derived as follows for a test with α≠90°, as per Di Fratta et al. [28]:

(8) $$\;\alpha_{\theta }=\tan^{-1}\left(\frac{{sin}^{2}\theta +\beta {cos}^{2}\theta }{\left(1-\beta \right)sin\theta cos\theta }\right)\;K_{x}=\frac{K_{\theta }\left({sin}^{2}\theta +\beta {cos}^{2}\theta \right)}{\beta }\;K_{y}=K_{\theta }\left({sin}^{2}\theta +\beta {cos}^{2}\theta \right)$$

where the in-plane permeability anisotropy β is defined as

$$\beta =\frac{K_{y}}{K_{x}}$$

Therefore, if the global directional permeability $K_{\theta }$ and two of ($\alpha_{\theta },\theta ,\beta$) are known, the principal permeability components $K_{x}$ and $K_{y}$ can be obtained. In Section 3, we describe an improved experimental method in which $K_{\theta }$, α, and θ can be obtained concurrently and the two principal in-plane permeability components can be derived according to Equation (6).

## 3. Materials and Methods

### 3.1. Materials

**Figure 3:**
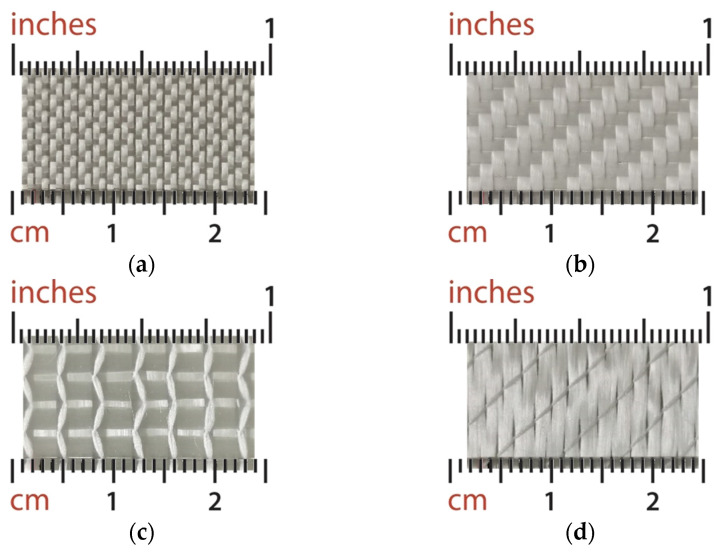
Fibrous fabrics investigated in this work: (**a**) Satin fabric; (**b**) Twill fabric; (**c**) Biaxial fabric EKB424; (**d**) Biaxial fabric EKB450.

Four glass-fiber fabrics in different woven patterns were investigated for the development and validation of the newly proposed concurrent unsaturated in-plane permeability characterization method. The woven structure of each fabric is shown in Figure 3. The fabrics were referred to as satin, twill, biaxial EKB424, and biaxial EKB450 in the rest of this paper. The areal weights of these fabrics are 220 $g/m^{2}$, 327 $g/m^{2}$, 424 $g/m^{2},$ and 450 $g/m^{2}$, respectively. Vinyl ester resin, Atlac^®^ 430 LV GT 250, provided by Jinling AOC Resins Co., Ltd. (Nanjing, China), was used as the test liquid. The viscosity of the resin was characterized with DV2T touch screen viscometer from AMETEK Brookfield (Middleboro, MA, USA) before each infusion experiment. Note that the viscosity of the resin is considered constant during the test since it was uncatalyzed. The resin was degassed in a vacuum chamber for 10 min to get rid of air bubbles.

### 3.2. Real-Time Flow Front Tracking and Image Processing

**Figure 4:**
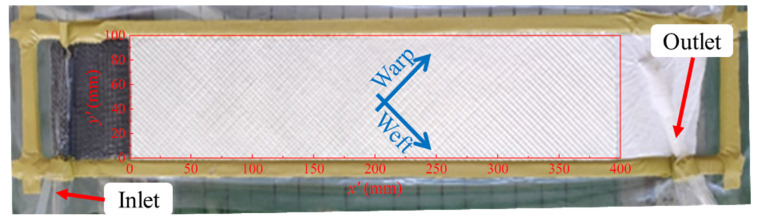
Test setup for concurrent in-plane permeability characterization.

The newly proposed concurrent unsaturated in-plane permeability test method, based on unidirectional injection, shares a similar experimental setup with the conventional unidirectional in-plane permeability test, as shown in Figure 4. The flow experiment set-up consists of a flexible vacuum bag as the top mold and a rigid lower mold. Lines are drawn every 2 cm on the lower mold for calibration of flow front positions. A fabric was cut into 10 cm × 40 cm rectangular plies and then assembled in the same direction as a 2-ply preform for testing. The preform was sealed between the lower mold and the vacuum bag perpendicularly to the vertical lines. The edges parallel to the flow direction were tightly sealed using sealant tape to eliminate edge effects. Attention should be paid to avoiding wrinkles or bridging on the vacuum bag side so that undesirable flow channels can be prevented. A spiral tube was used as a line injection port (inlet) and a stack of distribution medium was placed before the preform to create a fully developed one-dimensional flow. The outlet and the other end of the preform was connected by a breather to ensure the stability of the infusion pressure. The resin, driven by a constant vacuum pressure (9.2 × 10^4^ Pa maintained by 2XZ-2 vacuum pump, Zhejiang Taizhou Qiujing vacuum pump Co., Ltd., Taizhou, China), was infused into the mold cavity from left to right side after leakage checking.

A noteworthy aspect of this experimental setup is the integration of a novel flow front tracking velocimetry system, comprising an image acquisition device and an in-house real-time image processing code. The main limitation of optical imaging is the appropriate time resolution of the imaging device. This is not an issue in our case due to the slow flow front velocity typically observed in liquid composite molding, which is in the order of millimeters per minute. Hence, a 60 FPS (frames per second) webcam was utilized. This relieves, on the other hand, the bottleneck issues commonly encountered with optical imaging devices, that is, the high data rates that need to be streamed and stored in real-time.

**Figure 5:**
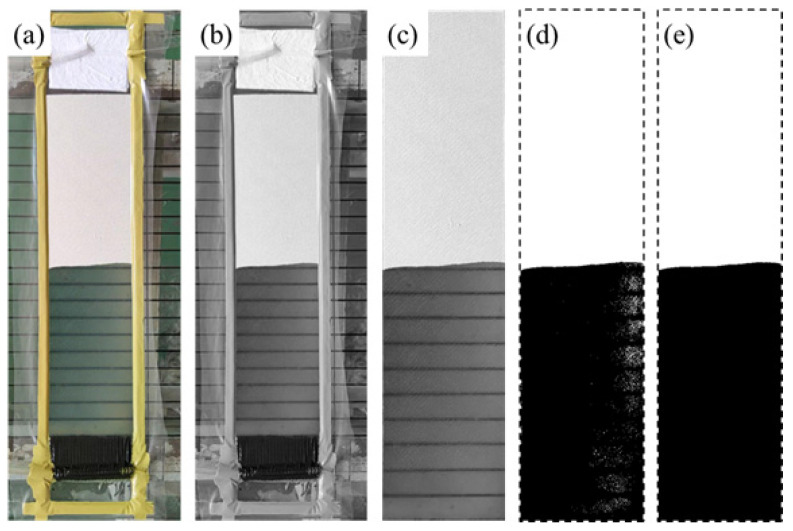
Procedure of real-time image processing and flow front detection: (**a**) field of view of the camera for RGB image acquisition; (**b**) grayscale conversion; (**c**) perspective correction and cropping; (**d**) segmentation of the saturated (black) and the unwetted (white) zones via thresholding; (**e**) denoising utilized median filter. The dashed lines were added to show the boundary of ROI in (**d**,**e**).

The code was implemented in MATLAB with the MATLAB Image Processing Toolbox as a basis. It allows for tracking and analysis of the flow in time and space. Figure 4 illustrates how a local coordinate $x^{\prime }-y^{\prime }$ was defined to allow the description of the local in-plane permeability to be more straightforward in the code implementation. The test device was placed at the center of the camera’s field of view (Figure 5a). RGB images were captured over time intervals of Δt and converted to grayscale for further processing. Only the region of preform was retained (see Figure 5c). It is important to ensure that the rectangular preform appears in its correct proportion and shape, free from any distortion caused by the camera angle. Therefore, perspective correction was performed prior to cropping. By thresholding, the saturated and unwetted zones were identified to locate the flow front. Figure 5d illustrates that denoise operations are essential to prevent pixel misclassification. The median filter provides satisfactory denoising results, as can be seen in Figure 5e. Afterward, each column of pixels along the test direction (referred to as Region of Interest or ROI hereafter) is isolated. The actual pixel size was calibrated according to the black parallel lines on the lower mold. In this way, the flow front $z_{ff}(t)$ position for each ROI can be accurately determined.

The principal axes of a permeability tensor usually coincide with the warp and weft directions of common textile reinforcements, thanks to their orthogonal weave structure. Thus, the angle θ defined in Figure 1 can be predetermined for those textiles by cutting the specimen at a given angle with respect to the warp direction. In this work, fabrics were cut and tested along the angle bisector of warp and weft directions (θ=45°, see Figure 4). Equation (6) reduces to the following equations:

(9) $$\tan\alpha_{45{}^{\circ}}=\frac{1+\beta }{1-\beta }\;\;K_{x}=\frac{\left(1+\beta \right)K_{45^{{}^{\circ}}}}{2\beta }\;\;K_{y}=\frac{\left(1+\beta \right)K_{45^{{}^{\circ}}}}{2}$$

As the flow front orientation angle $\alpha_{45^{{}^{\circ}}}$ and the evolution of flow front can be determined via the flow front tracking velocimetry system, the two in-plane principal permeability components ($K_{x}$ and $K_{y}$) and the local permeability $K_{l}$ can be determined simultaneously by a single test. The results were compared with those obtained by the radial injection method for validation.

## 4. Results and Discussions

### 4.1. Flow Front Angle

**Figure 6:**
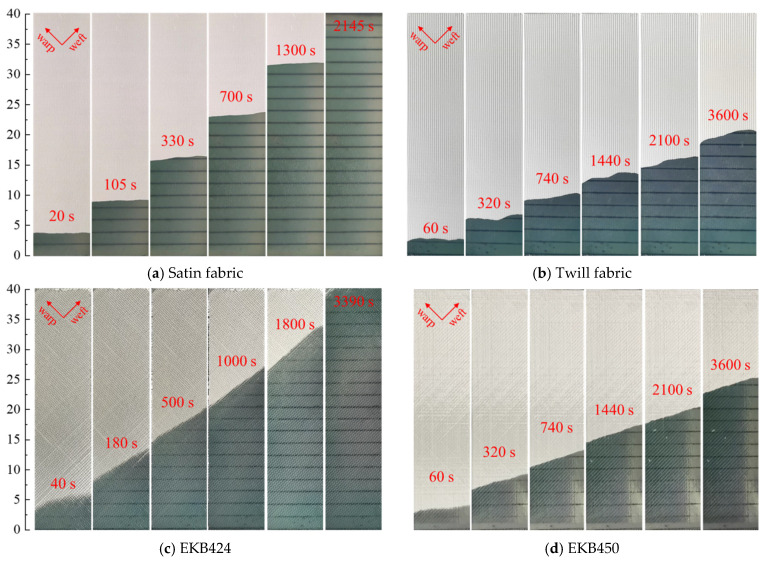
Experimental flow front profiles during unidirectional injection for different textiles.

Figure 6 depicts the evolution of the flow front for different textiles using the novel concurrent method. As illustrated in Figure 2b, let α denote the angle (≤90°) between the flow front profile and the flow direction, that is, the flow front angle. The flow front profiles differ significantly in terms of α for the four textiles, indicating the dependence on the textile structure. For instance, the flow front of satin fabric is almost perpendicular to the flow direction (α→90°), whereas biaxial textile EKB424 exhibits a noticeable angle change in the flow front with respect to the injection direction ( α≪90°). The flow front angles of the remaining two textiles lie between those of satin and biaxial EKB424 fabrics; however, they are also different from each other. This demonstrates experimentally that a unidirectional injection performed on a sample that deviates from its principal direction of the permeability tensor results in an inclined flow front.

**Figure 7:**
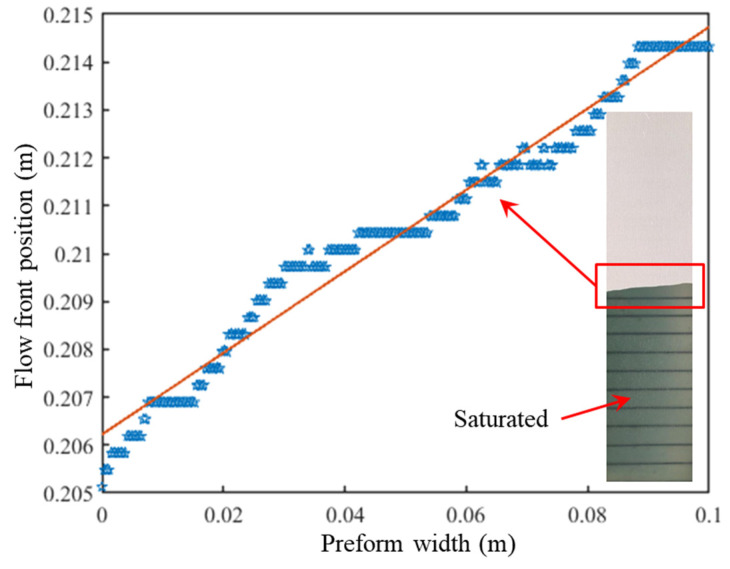
Flow front position and angle determination of satin fabric at 572 s.

The inset in Figure 7 shows the resin flow of satin fabric at 572 s. The pixels on the flow front are identified via the newly developed flow front tracking velocimetry system. The coordinates of these pixels are located accurately, with the black lines serving as calibration positions and plotted in Figure 7 as scatters. By using linear regression to approximate the flow front profile, the flow front angle α obtained at this moment is 85.13°. Tests on fabrics of the four different structures showed that the newly designed system can reliably obtain the position of each pixel on the flow front, thereby obtaining the corresponding flow front angle α in real-time.

**Figure 8:**
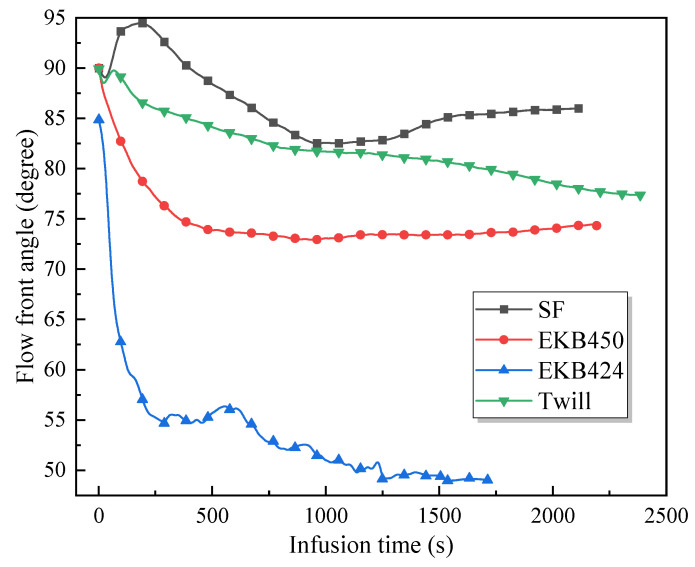
Flow front angle α as a function of infusion time for different textiles. The preforms were cut along the bisector of warp and weft direction.

A time interval of Δt=1 s is selected to capture the flow front, allowing for tracking the evolution of the flow front angle over infusion time. It strikes a balance between accurately capturing the dynamics of the flow front in the measurements and reducing the size of the data stream for real-time processing. The result is depicted in Figure 8. For all the textiles, the flow front angle undergoes an initial decrease, followed by a gradual stabilization with time. This decrease can be attributed to the dynamic nature of the fluid flow during the initial stage. The large pressure gradient at the initial stage results in high liquid flow velocity, induces unsteady flow behavior, and contributes to the significant changes observed in the flow front angle. However, as the infusion progresses, the flow gradually slows down, resulting in a more stable flow regime. This decrease in flow velocity contributes to the stabilization of the flow front angle. As the fluid reaches a quasi-steady state, the flow front angle tends to exhibit less variation and becomes more consistent over time.

It should be noted that despite the overall stability observed in the flow front angle during the late stages of infusion, small fluctuations may still be present. It can be attributed to various factors, among which is the spatial variability of textile microstructure resulting from the weaving and preforming processes. This intricate nature introduces local permeability variations. It may lead to numerous manufacturing defects if an infusion scheme is designed with global permeability [30]. In addition, the gap between tows in the EKB424 fabric creates fluid preferential flow channels, causing a substantial unsaturated zone behind the flow front compared to other fabrics. In this regard, digital image processing techniques have become more challenging, primarily due to the failure of thresholding segmentation techniques to correctly identify the flow fronts. A combination of image morphology operations, such as denoising, erosion, and dilation, is essential to address these challenges effectively. The flow front angles of satin, twill, EKB424, and EKB450 fabrics are approximately 86°, 78°, 58°, and 74°, respectively, after stabilization. As discussed in the next section, the difference in flow front angle mainly results from the permeability anisotropy of the fabrics.

### 4.2. Local in-Plane Permeability

**Figure 9:**
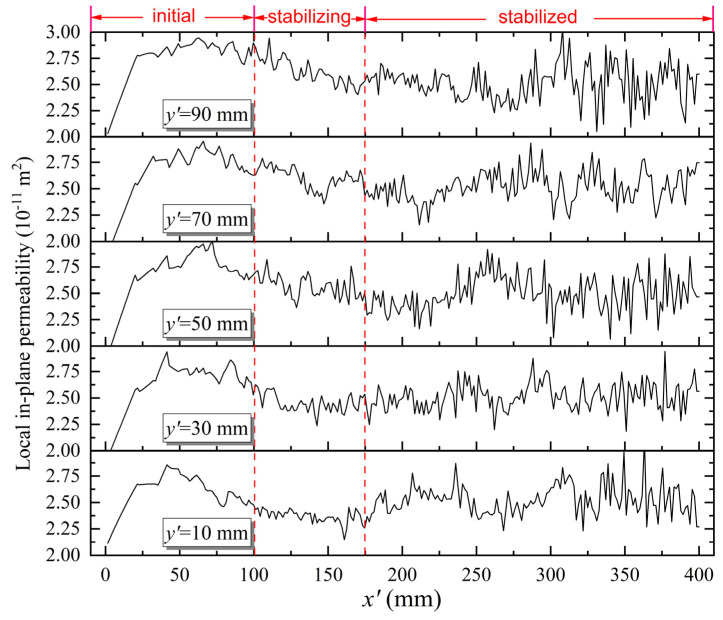
Local in-plane permeability distribution of satin fabric.

The local in-plane permeability was determined using Equation (4) in accordance with the methodology described. In the case of satin fabric, a total of 69,168 values was yielded via a test conducted with an imaging time interval of Δt=8 s. Among these values, 375 were discarded due to the interference of the inclined flow front with the outlet ($x^{\prime }=400$ mm, see Figure 4 for the definition of local coordinate $x^{\prime }-y^{\prime })$, which results in a change in the boundary conditions. Figure 9 depicts the measured local in-plane permeability of satin fabric along $x^{\prime }$ axis at $y^{\prime }=10,30,50,70$, and 90 mm, respectively. Noticeable variations were observed, indicating the inherent structural inhomogeneity and spatial variability of the fabric. This underscores the importance of considering the localized characteristics of permeability for robust infusion strategy design. On the other hand, the result demonstrates distinct stages. During the initial stage ($x^{\prime }<100$ mm), the test liquid was driven by a large pressure gradient. The liquid flow rate is notably high. Hence, the acquired data with an imaging time interval of 8 s are limited in capturing the full extent of flow fluctuations. As the test progresses and enters a more stable phase ($100<x^{\prime }<175$ mm), the results demonstrate a gradual convergence toward a stable stage ($x^{\prime }>175$ mm). During this stage, the fluctuations in the local in-plane permeability tend to cluster around a consistent mean value.

**Figure 10:**
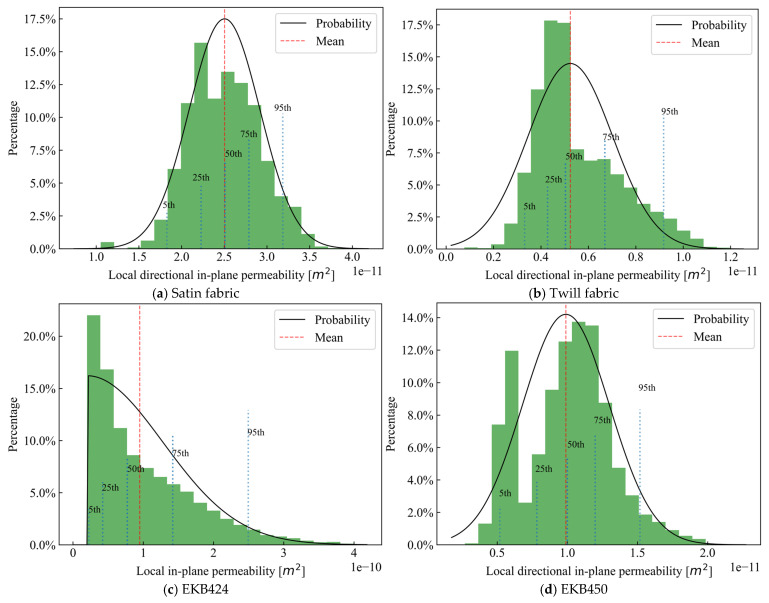
Permeability histogram of satin fabric in 0° direction.

**Figure 11:**
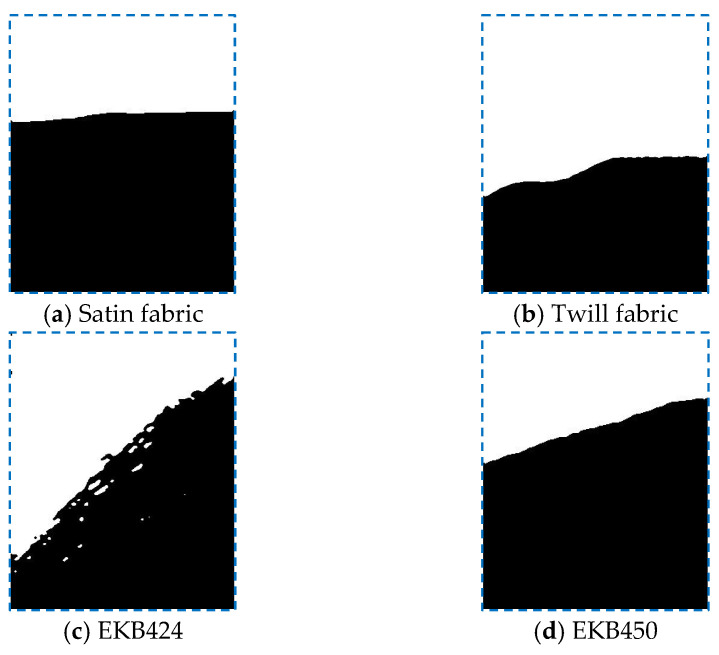
Typical shape of segmented flow front. Black for saturated zone, and white for dry fabrics.

Figure 10a depicts the frequency distribution of the local directional in-plane permeability values shown in Figure 9a on a histogram. The abscissa represents permeability values, while the ordinate represents the percentage of samples. The local in-plane permeability ranges between $1.0\times 10^{-11}$ and $4.0\times 10^{-11}\;m^{2}$. The mean value is $2.49\times 10^{-11}\;m^{2}$, which aligns with the 50th quantile. Toward the edges of the histogram, the frequency of samples decreases. The standard deviation (σ) of the dataset is $4.52\times 10^{-12}\;m^{2}$. Despite the right-skewness of the data (slightly higher concentrations in the right half of the histogram), they resemble closely a normal distribution pattern, as shown by the probability curve in Figure 10. The vertical blue lines represent the 5th, 25th, 50th, 75th, and 95th percentile values. As they provide additional insights into the data, a comprehensive understanding of the distribution of permeability and, consequently, the spatial variability of fabric microstructure are made possible. A similar pattern is also observed for the twill fabric and the EKB450 biaxial fabric, as depicted in Figure 10b,d, respectively. However, for the EKB424 biaxial fabric, a distinct half-normal distribution is evident, as illustrated in Figure 10c. The data, in this case, are concentrated toward smaller values, with a tail extending toward larger values. This observation could be attributed to the ambiguous boundary between the saturated and dry fabrics for EKB424 (see Figure 11), resulting from partial saturation behind the flow front. This partial saturation zone is also visually evident in Figure 6c. In contrast, for the other fabrics, a clear flow front can be easily segmented using image processing techniques, thereby avoiding such disturbances.

Note that despite obtaining all the data presented in Figure 9 and Figure 10 using an imaging time interval of Δt=8 s, a sensitivity analysis indicates that varying the time interval over a relatively wide range (from 1 s to 31 s) leads to fluctuations in the average local directional in-plane permeability ($K_{45}$) of less than 1%. This suggests that this method is not significantly influenced by the time scale.

### 4.3. Global in-Plane Permeability

**Figure 12:**
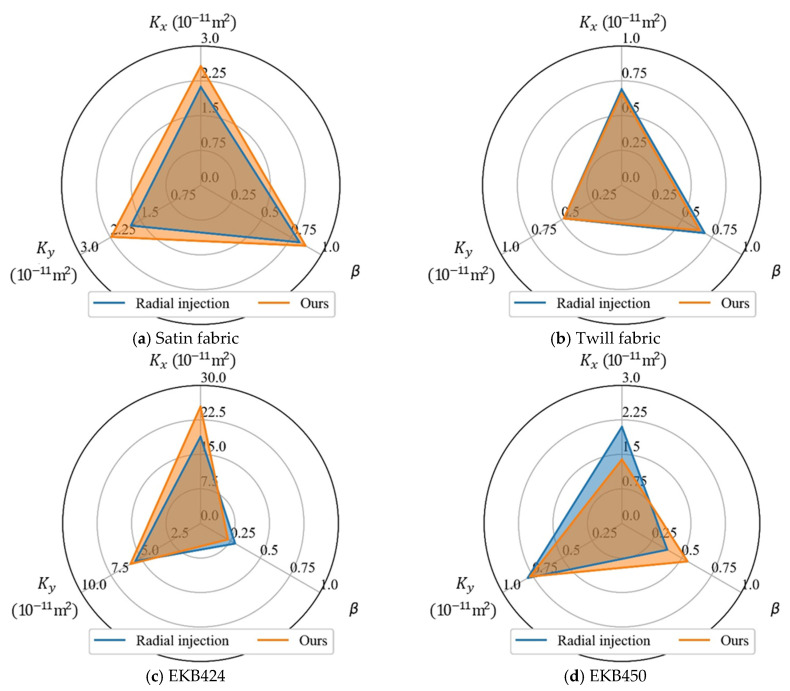
Global in-plane permeability ($K_{x}\;and\;K_{y}$) and permeability anisotropy (β) of fabrics measured by radial injection method and the newly proposed concurrent method.

**Figure A1:**
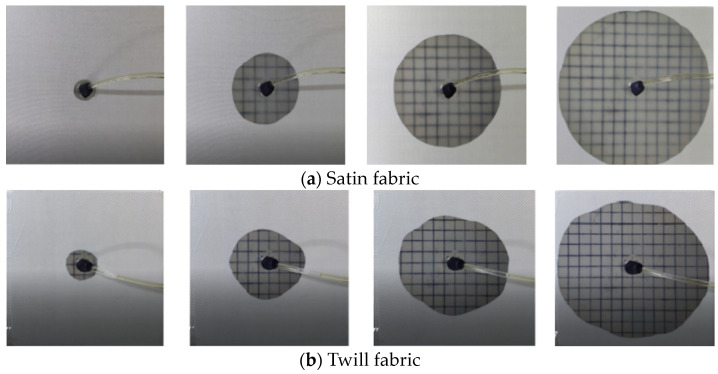

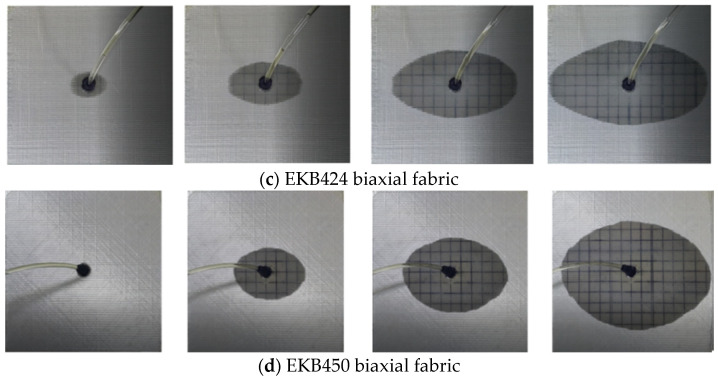
The filling state of textiles measured by radial injection. The distance between grid lines is 2 cm. Subplots (**a**–**d**) provide a qualitative comparison of flow front shapes for the textiles. The shape depends significantly on the woven architecture and always deviates from an idealized ellipse because of the inhomogeneous meso- and microstructure of actual textiles.

The global in-plane permeability of the four fabrics measured by the newly proposed concurrent method was compared with those measured by a commonly used radial injection method [15]. A sample of each fabric was cut into squares of 30 cm × 30 cm for the radial tests. As an injection port, a circular hole with a diameter of 23.9 mm was cut out in the center. The test liquid, vinyl ester resin, is driven by vacuum pressure ($9.2\times 10^{4}$ Pa). Figure A1 shows typical flow front shapes for the textiles (see Appendix A). The principal in-plane permeability $K_{x}$, $K_{y}$, and permeability anisotropy β of each textile obtained via both methods were compared, as shown in Figure 12. For satin and twill fabric, the global in-plane permeability and permeability anisotropy measured by the two methods are very close. As compared to radial injection, the newly developed method based on unidirectional injection requires fewer materials and simpler equipment. With the novel flow front tracking velocimetry system, it allows for high-throughput testing of in-plane permeability, which reflects the meso- and microstructure fluctuations of reinforcement fabrics. The test results of the latter two fabrics, however, deviated significantly from the baseline. It is primarily due to the partial impregnation behind the flow front, which makes it difficult to determine the location of the flow front. Therefore, improving the accuracy and reliability of the flow front-tracking velocimetry system is critical for the successful implementation of the proposed concurrent method.

### 4.4. Variability by Mesostructural Analysis

**Figure 13:**
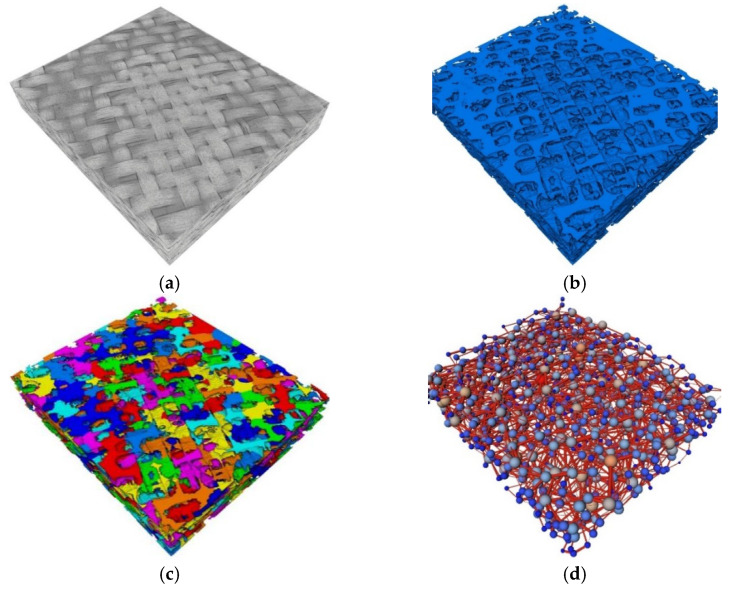
Volume imaging and analysis of textile mesostructure with Micro CT: (**a**) volume image of satin fabric; (**b**) segmentation of the mesopores with thresholding; (**c**) partitioning the mesopore space into individual subdomains with SNOW algorithm and represented with different colors; (**d**) pore network model.

The mesostructure of satin fabric was also analyzed with a volume imaging technique for validation purposes. Each subplot of Figure 13 represents a step of the analysis procedure. Figure 13a shows the volume image of the satin fabric acquired via micro-computed tomography (pixel size = 15.3 μm), providing a visualization of its mesostructure. The volume image shows a complex weave structure, with different yarns and interlacing patterns contributing to its spatial variability. In Figure 13b, the mesopore space (the gap between fiber tows) was segmented using thresholding. Note that the micropores inside fiber tows were neglected since they did not have a significant effect on the seepage property of the fabric. To analyze the geometric characteristics of the porous structure, a Pore Network Model (PNM) was reconstructed. The mesopore space was first split into individual subdomains with the SubNetwork of the Oversegmented Watershed (SNOW) algorithm [31]. These subdomains are shown in Figure 13c with different colors. The subdomains were modeled as spheres or cylinders in the PNM model so that the material can be represented as a network of interconnected pores, as shown in Figure 13d. It provides a simplified representation of the actual mesostructure.

**Figure 14:**
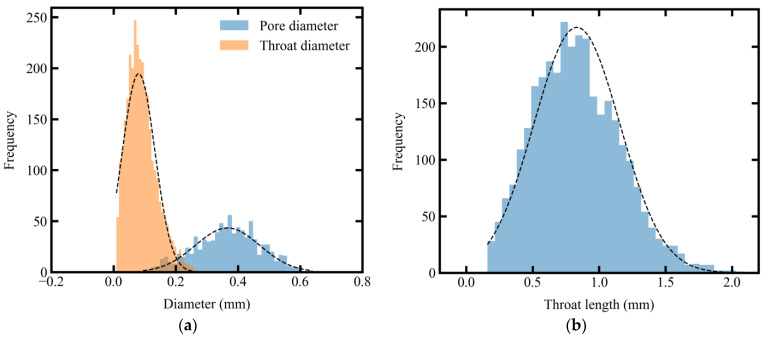
Measured distribution of (**a**) pore and throat diameter and (**b**) throat length for satin fabric.

As a simplified representation of actual fabrics, the PNM provides valuable insight into mesoscopic geometric characteristics. As illustrated in Figure 14, the spatial variability of the mesostructure was analyzed via the distribution of pore and throat size. The distribution of pore diameter, throat diameter, and throat length closely resemble the normal distribution. This agrees with the conclusion drawn from the stochastic analysis of local in-plane permeability. However, it should be noted that the tail of the distribution of throat diameter is truncated toward lower values. A similar phenomenon is observed for throat length, with the throat length exhibiting left-skewness and throat diameter right-skewness. This indicates a right-skewed distribution of throat flow capacity, considering that the fluid flow capacity through a porous medium is directly proportional to the square of throat diameter and inversely proportional to its length, as described by the Hagen–Poiseuille equation. The tendency for a local area to have a high flow capacity aligns with the distribution of local in-plane permeability, which shows a higher concentration of values on the side of high permeability (see Figure 10). This highlights the strong influence of geometrical variations on the fluid transport properties of textiles. The structure variability of fabrics indicates that the material possesses heterogeneous properties, which may cause non-uniform flow and lead to potential defects at the fabrication stage of polymer composites. Thus, characterizing fabric structure spatial variability is crucial to gain a better understanding of its fluid transport properties. Such insights can aid in designing optimal and efficient resin infusion strategies.

## 5. Conclusions

A comprehensive understanding of the permeability variability of reinforced textiles plays a crucial role in enhancing the efficiency and reliability of manufacturing processes for polymer composites. The proposed concurrent in-plane permeability test method addresses the challenges associated with textile structural distortions and dual scale flow, offering an efficient and cost-effective approach to simultaneously estimate structural variability and local and global in-plane permeability via a single test using unidirectional injection. The result can be leveraged to optimize resin infusion strategies by considering the spatial variability and uncertainty in numerical simulations rather than regarding permeability as a constant. In this way, the fault tolerance of the designed infusion strategy can be enhanced, contributing to defect-free manufacturing processes in real-life situations.

The following main observations are made: (1) The integration of real-time digital image processing substantially augments the capacity for data acquisition and processing and facilitates quantitative investigation of permeability variability through a high-throughput manner. By circumventing the flow perturbation induced by embedded sensors and mitigating the limitations associated with single-point monitoring, this non-intrusive approach enables a comprehensive assessment of local permeability variations. It permits the establishment of process boundaries within which the fabrication process can operate effectively and reliably; (2) Coupled with theoretical derivations, the proposed method enables the determination of the principal in-plane permeability components through a single experiment, while traditional unidirectional methods typically necessitate three unidirectional injection tests along different directions; (3) This method has the advantage of reducing the cost and time associated with volume imaging, such as magnetic resonance imaging or micro-computed tomography. Moreover, the proposed method provides valuable information on long-range textile structure variations, while the volume imaging technique is usually limited to the representative elementary volume level.

The present results work as a first step toward a high-throughput, cost-effective characterization methodology of in-plane permeability and spatial variability of textiles. Future work involves further enhancing the stability and reliability of the image processing system and exploring its applicability to various textile structures. Moreover, endeavors are being made in our team to establish a direct relationship between the local structure of fabrics and the spatial variations in permeability, which will be presented in separate works.

## Data Availability

All the data is available within the manuscript.

## Appendix A

This experimental setup for determining unsaturated in-plane permeability based on radial flow involves compressing a stack of textile layers between two rigid molds while maintaining a constant gap height between them. Afterward, a test fluid is injected through a central circular injection hole punched into the textile material (23.9 mm in diameter). In this manner, a two-dimensional flow pattern is generated. The flow front is tracked during this experiment. The test fluid is drawn into the mold through the injection hole by vacuum. Theoretically, the flow front should resemble an ellipse. As shown in Figure A1, there are, however, deviations from the ideal ellipse in practice. The flow front exhibits fluctuations induced by inherent variability present within the sample and its microstructure. These fluctuations are influenced by factors such as manufacturing or preforming. In addition, the weaving pattern shows a significant impact on textile transport behavior. The flow front of the satin fabric closely resembles a circle, indicating its isotropic nature. The remaining three textiles, however, exhibit elliptical flow fronts with varying circularity, indicating different levels of in-plane permeability anisotropy.
